# Ethics support for ethics support: the development of the Confidentiality Compass for dealing with moral challenges concerning (breaching) confidentiality in moral case deliberation

**DOI:** 10.1186/s12910-024-01039-7

**Published:** 2024-05-03

**Authors:** Wieke Ligtenberg, Margreet Stolper, Bert Molewijk

**Affiliations:** 1 Department Ethics, Law & Humanities, Amsterdam UMC, Amsterdam, The Netherlands; 2 Clinical Ethics Support, Center for Medical Ethics, Oslo, Norway; 3 Department Ethics, Law & Humanities, Amsterdam UMC, Ethics Support & Research Integrity, Amsterdam, The Netherlands

**Keywords:** Confidentiality, Breaching confidentiality, Moral case deliberation, Ethics support tools, Development process

## Abstract

**Background:**

Confidentiality is one of the central preconditions for clinical ethics support (CES). CES cases which generate moral questions for CES staff concerning (breaching) confidentiality of what has been discussed during CES can cause moral challenges. Currently, there seems to be no clear policy or guidance regarding how CES staff can or should deal with these moral challenges related to (not) breaching confidentiality within CES. Moral case deliberation is a specific kind of CES.

**Method:**

Based on experiences and research into MCD facilitators’ needs for ethics support in this regard, we jointly developed an ethics support tool for MCD facilitators: the Confidentiality Compass. This paper describes the iterative developmental process, including our theoretical viewpoints and reflections on characteristics of CES tools in general.

**Results:**

The content and goals of the ethics support tool, which contains four elements, is described. Part A is about providing information on the concept of confidentiality in MCD, part B is a moral compass with reflective questions, part C focuses on courses of action for careful handling of moral challenges related to confidentiality. Part D contains general lessons, best practices and tips for dealing with confidentiality in future cases.

**Conclusions:**

This paper concludes with providing some lessons-learned related to developing ethics support tools and some reflections on issues of quality and normativity of ethics support tools.

## Background

In the Netherlands, moral case deliberation (MCD) is an established form of clinical ethics support (CES), with a growing practice within health care and other domains [1–5]. In the past 15 years, trainers of the ethics support group of Amsterdam UMC trained approximately 1500 MCD facilitators, nationally and internationally. Facilitators learn by doing, during the training, and afterwards when they are practicing facilitating MCDs in their own context [6]. Whilst facilitating, MCD facilitators might experience moral challenges themselves. Occasionally, trained MCD facilitators consult the ethics support group within Amsterdam UMC (department Ethics, Law and Humanities) about moral challenges. One of these moral challenges concerns dealing with the concept and agreements of confidentiality in MCD. The commitment to confidentiality, for both participants and facilitators of MCD, is a central element of MCD. Confidentiality agreements are central in MCD to ensure the integrity and moral quality of CES. Confidentiality promotes safety among the participants in MCD and is a condition for free and open speech during the MCD sessions. Our previous qualitative research into the meanings of the concept of confidentiality in MCD shows how it is described differently by MCD facilitators. In definitions it is often associated with different concepts like privacy, trust and reliability. The content of what exactly should be kept confidential according to MCD facilitators also varies from details from the case, notes, decisions, participants, and aspects of the content and the process of the specific MCD session [7].

Yet, confidentiality agreements sometimes lead to moral challenges for participants, managers [8] and also MCD facilitators. When confidentiality agreements conflict with other interests and values related to the CES case, the moral question arises whether or not to breach confidentiality as MCD facilitator. It may occur, for example, in situations in which participants in MCD say something about how care is organized or carried out, which points to a possible risk for patients. It might also arise when illegal acts are discussed or considered during MCD, or when a manager of a department asks the MCD facilitator to provide information about the content and process of a MCD at which he/she him/herself was not present. Whereas the MCD itself offers participants ample opportunity to reflect upon moral questions, MCD facilitators are left empty-handed when they are facing such moral challenges themselves.

What exactly is (breaching) confidentiality within the context of MCD? Is the MCD facilitator allowed or obliged to breach confidentiality and in what situations? If so, what are appropriate reasons and possible alternatives to consider before actually breaching confidentiality? How do you make such a moral decision, and what is a morally right course of action after deciding to breach confidentiality? Who should you involve and what is the right timing? Situations and questions like these made us decide to start a project on these moral challenges faced by facilitators MCD and to explore whether a CES tool could be developed to help deal with them [9].

In this article we describe the process of developing an ethics support tool. First, we place this development in the context of a focus on confidentiality in health care in general and in ethics support in particular. We then present our theoretical viewpoints on clinical ethics support and ethics support tools, including the methods used. Next, we describe the development process of the Confidentiality Compass, followed by a presentation of the content and goals of the different parts that constitute the ethics support tool. In the discussion we will reflect upon normativity within the development process, and present additional thoughts on integrative ethics support and some lessons learned regarding the developmental process of CES tools in general.

### Codes of conduct or confidentiality guidelines in health care

Confidentiality is a widely used concept in daily health care in general. It is of great importance for trust in and accessibility of the health care system [10, 11]. For many professions, such as doctors, lawyers and social workers, the description of the concept of confidentiality and handling (breaching) confidentiality is formally regulated by legislation, codes of conduct or professional standards [12, 13]. These guidelines on handling confidentiality and certain codes of conduct have the function of prescribing and providing answers to general situations, about what is allowed and what is not. However, it often remains unclear how to act in specific situations.

We did not find, either in the Netherlands or internationally, documented viewpoints, research or guidelines for MCD facilitators regarding confidentiality specifically. However, for (bio)ethicists in general and for ethics consultants, there have been numerous attempts to produce a code of ethics in order to create clarity about the responsibilities of bioethicists [14] and ethics consultants [15, 16]. These codes of ethics contain sections on advocacy, privacy, confidentiality and trust, and describe how bioethicists should never exploit the information entrusted to them during their activities for personal gain. The sections describe how they should ‘always respect confidentiality’ – with certain exceptions, such as when it is required to share the information by law, or in case of significant harm to third parties. Before taking actions of disclosure, the bioethicist should first consult the persons who offered information in confidence [14]. Information (private, personal information, medical records, written summary of case consultation) should be respected by all ethics consultants and only ‘shared in accordance with standards of ethics, law and hospital policy’ [16]. The latter should be done discreetly, only to those who need to know and only the minimal amount of information necessary. It is recognized that the scope of confidentiality is often difficult to determine [15, 17]. In the German context, research was conducted in order to formulate recommendations on how to deal with confidentiality and data protection in clinical ethics consultation [18]. Some of these recommendations are based on specific preconditions in hospital organizations [19].

### Confidentiality guidelines for MCD facilitators

In the Dutch context there are no formal policy documents or normative guidelines for MCD facilitators on how to deal with moral challenges related to confidentiality. There is no specific viewpoint/policy or guidance in case the MCD facilitator is in the unique situation that he/she experiences a moral challenge regarding breaching or not breaching confidentiality. This leaves room for questions concerning the responsibilities of the MCD facilitator related to confidentiality. These questions are sometimes also related to the different positions and tasks of the MCD facilitator, taking into account that MCD facilitators often have other or multiple professional roles (e.g., nurse, doctor, teacher, policymaker) within one institution or organization. Also, in the training and certification of MCD facilitators no specific attention is paid to the theme of moral challenges related to confidentiality.

Based on some critical confidentiality incidents when offering MCD (e.g., when participants in MCD decided upon illegal actions or when external parties, like the Health Inspectorate, asked the MCD facilitator to report about the content of a MCD session, [7]) and because of the lack of guidance or policy, we started to develop an ethics support tool for moral challenges related to (breaching) confidentiality in MCD. At the same time the project aimed to protect the trustworthiness and quality of MCD (facilitators) and ethics support services as such, as dealing in a morally appropriate way with confidentiality issues in CES is strongly related to trust and quality.

### Incentives for developing an ethics support tool

Starting from the general aim to develop ‘an’ ethics support tool, the process as well as the content and form of the final ethics support tool were open from the start of the development process.

Clinical ethics support tools are relatively new in the field of clinical ethics support (CES). The development of tools is part of a movement that focuses on integrative ethics support (IES). IES is critical about more traditional forms of CES [20] and focuses on a) materializing insights from former CES activities for future use, and b) a stronger embeddedness and applicability of offering ethics support in the workplace and in work processes (instead of having to leave the workplace to go to a CES meeting).

There are only a few examples of ethics support tools in the literature. They vary from developing a moral compass tool for questions of client autonomy, to an ethics support tool supporting care providers in gender care with questions concerning the competency of young clients, and an ethics support tool to help individual patients with life-limiting illnesses to take action and guide them through ethical decision making [21–25].

Little is known about the *how* of ES tool development. Hartman et al. (2019) described four characteristics of an ethics support tool according to the pragmatic hermeneutic approach, some of which are also to be found in the final content of our developed ethics support tool. The theoretical presuppositions of the pragmatic hermeneutic approach were a part of the development process; we started with concrete moral challenges that were experienced in our MCD practice. During the data collection (in interviews and moral case deliberation sessions) we focused on their understanding of, and experiences related with, the relevant moral concepts and considerations. Moreover, we were keen to stimulate other perspectives and relevant contextual details that emerged during the process in order to stimulate critical thinking and putting users of the tool in some one’s other shoes.

However, every tool development process has its own characteristics. We do not aim to create one specific method for every ethics support tool development. Instead, we aim to learn from each (new) topic and its significance for the specific target group, as well to take in account the (new) context for which a specific ethics support tool is developed and embedded. The different purposes of an ethics support tool (being informative, reflective, focused on decision-making, providing normative guidance or practical suggestions, or adding referral to further knowledge sources) are also important to determine per development process. Furthermore, it is important to tailor the way the tool is designed to the use of the tool in a specific setting.

Understanding and conceptualizing an ethics support tool is implicitly or explicitly related to one’s theoretical understanding and presuppositions of ethics support and ethics support tools. Therefore, we will first present our theoretical viewpoints on clinical ethics support and ethics support tools, followed by a description of the methods used to develop the ethics support tool.

## Theoretical viewpoints on clinical ethics (support)

Moral case deliberation is based on a pragmatic-hermeneutical and dialogical view of ethics (support) [22, 26–28]. Characteristic of this view is that in the reflection process in which one tries to answer moral questions, both one's own experience and the experiences of others are essential for the moral learning. The starting points for moral case deliberation and moral learning are not theoretical concepts, but rather the multifaceted experiences of different involved stakeholders. Attention is paid to the specific context of moral questions and challenges. Furthermore, the perspectives of stakeholders involved must always be part of the reflection process; within a dialogue and exchange of views, the values ​​and norms related to perspectives involved are taken into consideration when answering a moral question. Within our dialogical view on ethics (support), dialogue is primarily understood as a vehicle for moral learning; a process to gain moral insights and answers.

When applied to the concept of confidentiality, this means that defining confidentiality is contextual. There will therefore not be one single clear norm or code of conduct for how to deal with moral challenges related to confidentiality. From other professional fields we know that even if concerns related to confidentiality are formally regulated (i.e., in codes of conduct or professional standards), moral questions about how to interpret and apply these regulations may still arise [29, 30]. And sometimes moral challenges are caused precisely *because of* existing legislation, codes of conduct and/or professional standards. Dealing with confidentiality it is often not a simple trade-off between laws and regulations, but rather a matter of carefully balancing ethical principles, values and ideas and considering their contextuality.

In our theoretical viewpoint on clinical ethics (support), ethical codes and guidelines are unlikely to provide sufficient normative guidance. A framework or code of conduct for dealing with confidentiality will not and cannot cover or provide normative guidance on the diversity of all (moral) challenges regarding confidentiality in different settings of ethics support. Perhaps they can function as a heuristic starting point, but in the end, you need a dialogical approach for reflection on this theme. In line with this, we are convinced that joint reflection leads to more insight and a better learning process, for the group and the individual, than solely using a descriptive tool.

Hermeneutic and dialogical ethics take the viewpoint that all parties involved give substance to the various meanings of the concept of confidentiality. In addition, there is an experiential component: even if there were clarity about what we mean by confidentiality in a given context, people can experience and value confidentiality differently. This experience may differ; both with regard to how important confidentiality is to them, and also with regard to what they feel if they believe that confidentiality has been violated or breached. These theoretical viewpoints imply that an ethics support tool should in some way take into account the local characteristics of the context, the experiences and viewpoints of the involved stakeholders, and should include a multi-perspective dialogue about what is considered morally appropriate (and what not) when confronted with moral challenges related to confidentiality. This dialogue usually takes place between different individuals such as during moral case deliberations. The multi-perspective dialogue in a group can also enriched by perspectives who are *not* present but still considered relevant for the case and moral question at hand [31]. This is also the case when an individual uses an ethics support tool: the tool urges the individual user of the tool to consider different and sometimes opposing perspectives on the moral question. In this way, the individual use of an ethics support tool can still stimulate an multi-perspective dialogue.

## Methods

### Participatory approach

The Confidentiality Compass is the result of an integration of questions and input from ethics support practice, scientific research, and insights from other normative and conceptual frameworks in the literature. From the start of the project, we aimed for a tool that would help MCD facilitators with the question how to deal with moral challenges concerning confidentiality. Based on our earlier research in this larger study [9], we aimed to develop an ethics support tool based on the values, needs and expectations of stakeholders. The research and development design was inspired by the responsive evaluation approach [32]. This approach seeks to give stakeholders a voice [21, 33]. The main group of stakeholders we involved in the development process of the tool were potential users, a diverse range of MCD facilitators with at least 5 years experience. We also included several experts, like a legal expert and a confidential counsellor of an academic hospital. Stakeholders participated continuously in the development process. This co-ownership and the process of co-creation fits with the dialogical approach to CES and MCD [21, 22]. Design and content of the Confidentiality Compass were not predefined, nor did we have any specific development steps in mind. From the start tool development took place in a joint and responsive process with the stakeholders, in an iterative process.

### Research methods

We made use of various research methods to collect the data. In the next section we elaborate on the iterative process of developing the Confidentiality Compass and how we – using the different research methods—shaped the different phases in gathering our data.

We started with a literature review on the concept of confidentiality and by seeking examples and questions from the practice of European clinical ethics experts through email. Secondly, we conducted semi-structured interviews with MCD facilitators (*n* = 4) and experts who work with confidentiality in health care (*n* = 2). Besides traditional qualitative research methods, we also used ethics support activities themselves as a method for data collection.

In this study, we organized MCD sessions on moral challenges faced by MCD facilitators with the MCD facilitators themselves, and used these as a source of data for our research. Using MCD sessions as a research method is an unconventional practice. MCD is often part of research to study the content and processes of MCD retrospectively [3, 34, 35], not as a research method to collect data. In our project we studied the variety of beliefs, values and principles of the participants concerning moral challenges related to confidentiality, as well the variety of considered options for actions when (not) breaching confidentiality. Given these study aims, a structured reflection via MCD fitted well as a research method since it gave us the opportunity to study and reflect more in-depth and more systematically the motives, beliefs, values, norms and moral reasoning from MCD facilitators (as MCD participants) regarding (breaching) confidentiality. The MCD sessions were therefore purposefully organized by the researchers and the case-owner was invited to share his case based on its relevance for the topic of our research. Analyzing the content and process of an MCD session on confidentiality helped us to elaborate on insights and lessons learned from concrete experiences of MCD facilitators and how their views developed during the MCD dialogue. The MCDs were facilitated by two researchers in an online session; both times the dilemma method was used to structure the conversation [31]. To support the online interactive sessions and the joint learning process of the group, we used different interactive tools. For example, we used the Mind map program Mindmeister and Google Jamboard to stimulate the joint brainstorming and to secure as much input as possible. Based on the verbal input during the sessions and responses on Jamboard, a summary was made of each session, which was used as research data as well.

We also conducted two focus groups. During the first online focus group, with MCD facilitators from different professional backgrounds, we discussed our results from the literature review, interviews and MCD sessions. During the second focus group we piloted a draft version of the tool. We presented different parts of the tool and requested feedback regarding the content of the tool. Next, participants in the focus group practiced with the draft tool and provided initial reflections about its usefulness and applicability.

During different pilots of the tool, we gathered input and experiences from practice by means of thinking aloud interviews with MCD facilitators and brainstorm input from practice; e.g., parts of the tool were also presented and tested in practice during two peer-review sessions with MCD facilitators and two workshops at a conference for ethics support staff (NEON). During the workshop we discussed the topic of confidentiality and contemplated some of the reflective questions. Participants provided feedback on which questions were supportive for their personal moral challenge and which were not. Finally, we presented the tool in peer review meetings with MCD facilitators. The latter provided more insights from MCD facilitators with different traditions, professional backgrounds and levels of experience, which was useful to further refine the draft tool. The sessions provided the opportunity to discuss the tool collectively and collect instant feedback in the discussion among potential users.

### Research ethics

The Ethical Review Board of Amsterdam UMC was informed about the field studies. Ethics approval was not needed according to the Dutch Medical Research Involving Human Subjects Act (WMO).

All methods were performed in accordance with the relevant guidelines and regulations. Written informed consent was obtained from all participants and participation was voluntary and based on informed consent. Before the interview, MCD or focus group participants received an information letter with information about the study. This included information emphasizing the voluntariness of their participation, the possibility to withdraw from the survey study without giving reasons, and anonymity of the data.

## PART I. The iterative process of developing the ethics support tool

Although the content and form of the tool were developed in an iterative process, we can distinguish various phases in the research and development process. In the first phase we examined the concept of confidentiality in MCD and identified moral challenges of MCD facilitators and their specific needs for support in situations of doubt regarding confidentiality through literature review, interviews, MCD sessions and a focus group. Based on the results from those research activities, we developed an initial draft version of the ethics support tool, which was adapted into the final version in an iterative process through moments of tryout and evaluation. For the latter two workshops and some thinking aloud interviews were organized. Before presenting the final version of the Confidentiality Compass, we will first describe the different steps of the iterative development process.

1. Conceptual clarification and identifying moral questions

In the first phase we started to explore the concept of confidentiality. From the interviews and literature review it was not immediately clear how the concept of confidentiality was used during concrete ethics support activities. There were no clear guidelines or suggested courses of action in practice when confidentiality in CES became an moral issue. This resulted in a range from facilitators that were more confident in how to handle confidentiality questions into others that remained more insecure about how to handle certain cases in practice since they lacked some guidance. In addition, we approached European ethics support colleagues and asked them about their experiences and opinions. Some of them reported examples of pressing cases in which confidentiality of ethics support was at stake and described how they handled these within their institutions. Others declared that existing guidelines or agreements within the regular health care system (i.e. not specifically ethics support) were sufficient for them to provide clarity in case of confidentiality questions. None of the European ethics support colleagues referred to policy or guidelines especially developed for ethics support staff. Furthermore, to gain more in-depth insights about the role of confidentiality in health care in general, we decided to involve and interview two experts with broad experience in dealing with confidentiality in the health care context. All these steps helped us to gain insight into the concept of confidentiality and the moral questions about breaching confidentiality in practice from different perspectives [9].

2. Identifying specific needs for ethics support

In the interviews and in two focus groups we identified the needs for support with questions concerning confidentiality. In the first focus groups we focused on the needs and expectations of MCD facilitators concerning support and guidelines. These needs were diverse, varying from a need for information on specific questions and situations, a need for individual reflection and interpersonal reflection, and a need for specific policy or guidance on courses of actions for how to deal with confidentiality issues [9]. A MCD facilitator indicated that a peer-review group or consultancy with senior facilitators in which she could discuss questions and courses of action in a specific situations would be beneficial. She mentioned the need of ‘*something that provides support, so you don't have to make all choices yourself*’. Another MCD facilitator wanted more clarity on how to introduce the importance of confidentiality at the beginning of an MCD session. ‘*I would like more clarity about the agreements you make at the start of an MCD. I need something that helps to clarify the concept and meaning of confidentiality, but also one's own position as a facilitator related to the topic of confidentiality*.’ Others mentioned they wanted\needed\wished for something that can help them facilitate a conversation on confidentiality to increase awareness of the topic among the group. ‘*I am looking for input have a conversation in which one can discover that everyone views it differently, a conversation that ultimately increases awareness about confidentiality. I need something that shines light on things that may play a role, without being directive.*’ The diversity in needs inspired us to design one tool with different parts, each with a different goal, such as informing, reflection and instruction.

3. Determining the characteristics of the CES tool

We established some general goals for the tool, based on the empirical data and our own view and experience with ethics support tools; the tool should support reflection and create awareness about confidentiality. In this way it should contribute to the professionalization of moral case deliberation as a practice, and it should protect and support individual MCD facilitators.

During the process of data collection as well as designing the tool, we as researchers had many discussions about the content and design of the individual parts (informative, reflective, prescriptive etc.) of the tool. Initially, we aimed for a general reflective tool. However, the interviews variety in the knowledge about the various conceptions of confidentiality (in moral case deliberation) among MCD facilitators. For example, the concept of (breaching) confidentiality was interpreted in different ways by different MCD facilitators, from 'bringing in colleagues that are not directly involved in a specific case' (which was considered breaching confidentiality) to 'sharing information with your partner at home' (which was not considered a breach of confidentiality). This changed our focus from a reflective to a more informative tool. Ultimately, it became an integrated form of an informative—reflective tool. This was a process of constantly matching the needs and values from practice, with our view on features of such a specific tool, and realigning this with our general vision of an ethics support tool.

4. Presentation and discussion of the first draft of the CES tool

Based on the gathered empirical data a first draft of the tool (text only) was developed. One of the issues in the process of writing the first draft was the structure and position of the different reflection questions in the tool, as they could be linked to multiple parts of the tool. Furthermore, on the one hand we aimed for a tool in line with our theoretical viewpoints on CES and CES tools: an explorative and dialogical tool. On the other hand, we wanted to offer more than just a guided reflection following the—for facilitators MCD—well known-steps of MCD. We aimed for an extra level of reflection and sharing by including more content from our study. We ultimately decided to connect the reflection questions to the different themes and concepts that emerged during data gathering (the description of section B includes examples of themes and concepts such as professionalism, reliability, honesty and transparency). Next, we decided to structure the reflective part of the tool following the process steps of MCD because it follows a well-thought structured reflection process that leads to more in-depth reflection [31] and which are recognizable for MCD facilitators, the main users of the tool.

5. Determining the design of the CES tool

We discussed and considered different formats for the tool, e.g., a website, a dilemma game, a podcast or expert videos. We also discussed different target groups besides MCD facilitators, e.g., other professional groups, participants in MCD, managers and policy makers. Based on practical and financial reasons we decided in the end on an interactive PDF, with a main focus on MCD facilitators, but also to be used by other ethics support staff. The features of an interactive PDF allow for different elements of text, as well a kind of interaction with the user when he/she writes down answers on reflective questions. We also considered the accessibility of the tool; an interactive PDF can be easily accessed through, e.g., a website and can be downloaded and saved on a computer. This will make it easy to use and distribute.

6. Tryout rounds

The tool text was presented to different respondents for input in two iterative cycles. The first draft was presented to three experienced MCD facilitators in a thinking aloud interview. During these interviews the respondents reported us step by step how they would use the tool whilst reflecting on a personal case or moral question concerning confidentiality. The input from the three potential users involved suggestions to shorten sentences and paragraphs, to connect clearly the different elements of the tool, and to highlight more the flexibility of the tool e.g. using only the parts of the tool that are needed in a specific situation. Furthermore, they suggested to indicate clearer the intended audience and insert some examples to illustrate the more informative parts. Finally, the examples should be more diverse in order to make the tool also relevant for MCD facilitators working in other context, outside health care.

The combination of input from the thinking aloud interviews and the insights from the researchers led to adjustments to different parts of the tool. This resulted in improved understandability and readability of the text. We achieved this by using more accessible (less academic) language and shortening some parts of the tool. Furthermore, some changes were made to the informative parts by introducing a distinction between compulsory reading material as a general introduction, and optional in-depth readings. Furthermore, based on the feedback from research participants, the researchers realized that, apart from questions on values, there was a lack of reflective questions about virtues and weighing principles. We therefore added some virtues and principles in the reflective parts, to make the reflection more personal. These questions provoke reflection on personal characteristics and encourage the user to think about ‘being a good MCD facilitator’. Not only reflecting on idealistic or hypothetical values or principles that matter in the specific situation, but also on the question: what does being a good MCD facilitator mean to me? These elements from virtue ethics are also used in other forms of ethics support [36].

Before putting the different elements together in an interactive PDF, three experienced MCD facilitators (different from the ones that did the thinking aloud interviews) read through the modified text again and made some final remarks. These were mainly textual. The respondents asked for ways to interact and space to write down their thoughts while using the tool. Changes were made to different parts to make the tool more interactive. Answer options with the opportunity to write down reasoning and arguments were included to achieve an active learning process for users.

7. Fine-tuning content, goals and design

For the final design of the tool, the third iterative cycle, we collaborated with a graphic designer and worked together on the interactive PDF. Some elements needed to be adjusted to the format of an interactive PDF, e.g., texts were again shortened. We differentiated compulsory and optional parts through visual elements (i.e., elements that are not visible initially). A draft of the ‘online’ tool design was presented to two potential users (i.e., MCD facilitators)—that were not involved in previous steps of the development process—with questions about usability and clarity. Remarks were made to certain headings and interactive fields in the tool. They did suggestions to present certain concepts or questions clearer within their context. Their feedback led to some final changes to the tool: 1) every individual part in the tool now starts with a short introduction and description of the goal, 2) we added a user manual that describes how to use the parts individually and the option to use only parts that are relevant for the user, and 3) we included some quotes and added some visual elements to improve the readability of the tool.

## PART II. Presentation of the Confidentiality Compass

Following the above-mentioned needs for support and based on our viewpoints on CES tools, we divided the tool into 4 parts: Parts A, B, C and D. Part A provides information and aims to raise awareness of the multifaceted concept of confidentiality in MCD. Part B provides several reflection questions that aim to foster a better understanding of (the origin of) moral doubts and of values at stake regarding breaching confidentiality in a specific case. The goal of part B is to formulate an answer to the question whether to breach or to keep confidentiality. Part C also provides reflection questions and aims to foster reflection, but with a specific focus on types of actions after the decision to breach confidentiality or not. Part C provides substance to the question: once I have made my decision, how do I act in the right way? Part D contains guidance for practice: some general lessons, best practices and tips and suggestions for actions for general users. The first slides of the interactive PDF provide instructions for usage, e.g., an explanation that the different parts can be used independently, and the user is not obliged to read the texts and questions in a particular order.

In the next paragraph we will introduce in more detail the content of the four parts of the tool, including some examples.

### Part A: conceptual clarity: what is (a breach of) confidentiality (in moral case deliberation)?

During the first phase of developing the tool, several research participants expressed the need for conceptual clarification of confidentiality. Part A, therefore, explains the multifaceted concept of confidentiality without giving a universal definition. What does confidentiality mean in the context of MCD? Which definitions are used by MCD facilitators? This section explores different elements and concepts of confidentiality. In addition, this part identifies the importance of confidentiality and its role in MCD. After completing part A, the MCD facilitator has more insight into when confidentiality is relevant, a better understanding of the different interpretations of the concept and increased awareness of the moral dimensions of confidentiality.

### Part B: moral compass

Part B is an interactive part promoting moral (self) reflection through reflective questions and opportunities for answers. In this part, which can be seen as a moral compass, the MCD facilitator reflects on the specific context of a case (situation) in which moral challenges related to confidentiality arise. He/she follows a stepwise process, each step with a specific purpose:

1. Clarifying the situation and the experienced moral question. This step is about obtaining an overview of the relevant facts in the case and clarifying the experienced doubts in order to formulate the moral question(s).
2. Collecting values ​​and norms for different and sometimes contradictory answers to the moral question(s). This step covers a few specific values and thematic domains that emerged from our data. For example, relationships may play a role, or different responsibilities from those involved, or the value that needs to be protected (Table 1). This step ends with some closing questions and answer options regarding values and norms in the specific case.
3. Deliberating and weighing. In this step the user is supported in making a decision with additional questions to help weighing the relevant e.g., themes or values at stake. This step also contains questions about the considered course(s) of action, including weighing principles like subsidiarity, proportionality and effectiveness (Table 2).
4. Decision-making. This step offers the opportunity to answer the moral question(s) and indicate core values. It contains questions about relevant virtues (Table 3) and what it means to be a ‘good MCD facilitator’.

**Table 1:** Values ​​and norms

Examples of values: loyalty, professionalism, safety, reliability, justice, honesty, transparency, protection
Examples of questions about values: **Responsibilities**
• Looking at the confidentiality agreements made with the team and/or the organization that organizes the moral case deliberation: What do the agreements mean for *your* responsibilities in this situation?
• Apart from any possible agreements made with the team and\or organization: what do you see as your responsibility in your role as MCD facilitator? In what way are you responsible or should you (not) feel any responsibility?
• Who else bears responsibilities regarding keeping or breaching confidentiality in this situation? Why is it your moral question?
• What is your responsibility as an MCD facilitator in relation to your other functions/roles?

**Table 2:** Weighing principles

**Proportionality** (Are your actions proportionate, considering the consequences?)
• Looking at your considerations, are they in proportion to the seriousness of the situation and the possible impact of your decision? In other words: is your action too big or too small, in view of what is at stake?
• How do the consequences of breaching confidentiality relate to the consequences of not breaching it?
**Subsidiarity**: (Is this the best way? Are there other ways?)
• What is the damage if you do nothing? Can you prevent this by doing something else, without breaching confidentiality?
• Do you have a clear idea of relevant alternatives?
**Effectiveness**: (Does your action achieve the objective?)
• How do you achieve what you have in mind with your final decision and the accompanying action?
• Which action has priority, what can wait or be postponed?

**Table 3:** Virtues

The following questions are inspired by the four cardinal virtues and can help users to evaluate their decision
Courage:
• What courage is needed here?
• What has to be overcome?
Prudence:
• What should we consider more closely?
• What do I have to face?
• What do I want to protect?
Temperance:
• What do I have to specify?
Justice:
• What does this mean for mutual relationships?
• What is needed to do justice to those involved?

### Part C: reflecting on actions; careful handling of confidentiality

In our investigation of needs [9], research participants were eager to think about different (escalation) steps and ways to reflect on the question: how do you deal with (breaching) confidentiality in the right way? There was also a need for attention to limiting the negative consequences of actions and take into account what kind of aftercare would be preferable.

Part C thus focuses on supporting MCD facilitators in taking actions that suit best the moral decision following from Part B. Part C reflects on the question: considering my action(s), how do I do this in the right way? (Table 4). It is important to know that the information in part C does not provide clear answers or prescribed courses of ‘right’ actions but supports the process of determining the right action. It describes three main courses of action, with different sub actions and includes a few questions about mitigating the negative consequences of the considered action as much as possible. In this way part C also deals with aftercare: understanding the possible disadvantages of your decision and how to keep them to a minimum.

**Table 4:** Actions

Purpose of this step: this is about reflecting on suggestions, not about prescribing certain actions
Once you have made a decision on whether or not to breach confidentiality, there are several possible scenarios, follow-up questions, and follow-up actions
I haven't decided yet, I will…
• … talk to …
• Someone from the ethics support department
• My manager
• A confidential advisor
• A colleague MCD facilitator
• alternative: ……… [write down what you will do]
• … discuss it during a peer meeting or a training moment with …
• … plan a moral case deliberation about this with my colleagues
• alternative: ……… [write down what you will do]
I have decided (for now) not to breach confidentiality, I will …
• … try alternative actions first to avoid having to breach confidentiality, namely …
• … do nothing for the time being and wait to see what happens
• … first talk to …
• participant X
• the whole MCD group
• the originator of this moral deliberation
• alternative: ……… [write down what you will do]
I have decided to breach confidentiality
What?
• Try to share as little information as possible, but enough to achieve your goal
• alternative: ……… [write down what you will do]
Who?
• Try to provide as little information as possible to only a few people, enough to achieve your goal
• Try not to pass on information too high in the hierarchy; first closest to the people in question
• alternative: ……… [write down what you will do]
Informing
• Who do you inform about your action(s)?
• Your manager and/or the ethics support coordinator
• The MCD participants
▪ About what I'm going to do (or have done)
▪ About how I came to this decision
• alternative: ……… [write down what you will do]
Justifying
• See how you should record and report things to justify your choice and action
• Consult with colleagues, ethics support coordinator and/or manager what you can learn from this situation and what could be done differently to prevent this in the future
• alternative: ……… [write down what you will do]

### Part D: general lessons, best practices and tips

Because of the aim of professionalization of MCD and MCD facilitators, part D contains a number of general questions and recommendations to support the skills of MCD facilitators in (future) situations of confidentiality questions (Table 5). In this way the tool does more than just react to a specific case and think about possible related actions. The final part also aims to be proactive with a focus on early de-escalation.

**Table 5:** General lessons and tips

• What have you learned about yourself as a facilitator by going through the questions in this ethics support tool?
• What lesson do you take away regarding dealing (better) with confidentiality in the future? What can other MCD facilitators and ethics support colleagues learn from this?
• What would you do differently next time based on your experiences?

Furthermore, a number of proactive tips are included to better deal with confidentiality next time. And finally, it contains three descriptions of *best practices;* anonymized stories about handling questions concerning confidentiality in MCD (illegal actions announced; well-being of the team at stake; external investigation and MCD), with lessons learned.

## PART III. Lessons learned from developing an ethics support tool

Considering a lack of literature on *how* to develop an ethics support tool, we will now share the lessons we learned during the development process.

Developing the Confidentiality Compass did not only provide us with insights into the content of confidentiality in MCD, but also with a number of lessons learned related to tool-development which may be helpful to future processes of developing ethics support tools. We will discuss these lessons learned with regard to clarifying the concept, additional tool characteristics and reflections on normativity.

### Insights into clarifying the concept

When developing an ethics support tool, it is important to continuously monitor and check which questions are relevant in practice in relation to the theme of the tool. Confidentiality in MCD turned out to be a much more complex theme than we initially thought. Moral challenges related to confidentiality appeared to be more latently present among MCD facilitators. While we presumed challenges to occur in extreme and pressing cases in which breaching confidentiality is an urgent and stressing issue, the phase of conceptual clarification showed that research participants had a broader view on confidentiality and its implications for daily MCD practice. For example, research participants considered the conditionality of confidentiality; how confidentiality is a precondition for the dialogue about moral issues. They also introduced themes such as confidential reporting: how to handle notes and reports from MCD confidentially, and perceived safety in MCD: how do you ensure MCD as a safe space for moral reasoning. By highlighting the various meanings and descriptions of this concept in the research and development process, mutual awareness about this theme was created throughout the project.

We observed a diversity in casuistry and moral challenges from practice. Even research participants who had not yet experienced concrete issues concerning breaching confidentiality in practice provided possible moral questions about confidentiality and its meaning in MCD practice. The diversity in experiences and questions made us decide to distinguish different goals for the ethics support tool, one of which should be providing information on the concept and its relevance for MCD practice.

In conclusion, we have learned that during the tool development process we were already facilitating awareness and professionalization in the future users. When developing an ethics support tool, do not focus only on the initial problem which is central as the antecedent of developing the tool, but be open minded and attentive to the opportunities of raising awareness throughout the development process.

## Discussion

In this article we present the results of an iterative development process of an ethics support tool concerning moral challenges related to (breaching) confidentiality in MCD. We described the different phases, considerations and changes to the design of the Confidentiality Compass. Furthermore, we presented the content of the different parts of the tool including their goals, such as providing information, fostering reflection and showing guidance, and provided examples of questions and text elements of the tool. In the discussion we will describe some lessons learned about CES tool characteristics and we will also reflect upon and discuss developments like integrative ethics support, and the strengths and limitations of this research.

### Lessons learned about CES tool characteristics

Looking back at the characteristics of an ethics support tool according to the pragmatic hermeneutic approach as described by Hartman et al. (2019), we may conclude that the tool we have developed only partially meets the four characteristics [22]. Part B, with reflective questions, is focused on an urgent moral challenge and takes a problem that has been experienced as the starting point. However, the other parts of the Confidentiality Compass are informative and provide guidance on a more general level and can be beneficial to users even if they do not experience an urgent moral challenge. As to the second characteristic, that focuses on inquiry into the moral concepts, questions and routines within the lived experience of the tool user, we did include some predefined moral concepts. However, at the same time we included reflection questions with the aim to inspire users and to support them in reflecting upon relevant concepts of confidentiality. By doing so we combined both ‘providing knowledge’ and ‘promoting reflection’ as parts of a moral learning process. The third characteristic, which is about the focus on moral learning by exploring other perspectives, is achieved through relational reflective questions. The Confidentiality Compass is, however, primarily developed for individual use and personal reflection. Furthermore, a tool focusing on confidentiality may even be at odds with promoting moral learning. In some situations, maintaining confidentiality may hinder moral learning of teams or organizations, e.g., when results from MCD cannot be shared in detail. This in turn raises new questions, such as 'to what extent should breaching confidentiality serve the higher purpose of “learning as an organization”’? Another moral question then is: to what extent may one share the outcomes of MCD for the further development and moral learning of specific situations? The fourth characteristic of incorporating contextual details, such as norms and values of different stakeholders, but also rules and regulations, is also central in the Confidentiality Compass.

Based on our experiences we suggest the following additional characteristics for developing an ethics support tool. When a certain concept is not yet defined in a specific context or when there are multiple ways to interpret and apply this concept, it can be useful to include informative elements in an ethics support tool. By doing so you not only promote moral reflection but also provide knowledge, an indispensable part of moral learning. Furthermore, we added a description of best practices, which provides room for sharing good examples to inspire the users of the tool. Another element we added in the Confidentiality Compass is the provision of a variety of proposed courses of actions. By doing so we aimed to provide some directions without being normative and setting or prescribing a standard. With certain themes, especially relatively unknown or unexplored themes, rather than solely reflecting on values and norms, it might be useful to provide a little more guidance on actions. Lastly, in addition to describing some values and norms and asking for additional ones, which is common in traditional ethics support and previous examples of ethics support tools, we also presented moral questions related to possible virtues and weighing principles like subsidiarity, proportionality and effectiveness. The principles guide the users in making decisions and deciding on the urgency of certain actions. We took inspiration from different ethical traditions, such as virtue ethics, care ethics and consequentialism, and aimed for a more integrative approach of different backgrounds. Moreover, we aimed to encourage users to not only reflect on values and norms but also embody the virtues they consider important, and foster learning how to apply all this in specific situations and to develop the related qualities themselves [37, 38].

### Normativity and applicability

During the process of developing the Confidentiality Compass, there were several moments when we reflected upon questions about our normativity, related both to our role as researchers and developers and to the content and design of the tool.

So far, CES tools were developed for health care professionals [21, 22, 24]. In these examples, the developers were mostly CES staff that had no specific normative aim or goal in developing the tool itself, nor in the content or design of the tool. They co-created a tool aiming to support health care professionals in dealing with specific moral issue or questions. What we did in this project is different in several ways. First, the Confidentiality Compass is developed for CES staff themselves as the target group. Furthermore, as developers we considered this CES tool a form of professionalization of our professional domain: CES. Through developing 'ethics support for ethics support', we also aimed to contribute to the quality of MCD facilitators, which implicitly indicates what we consider to be the quality and expertise of MCD facilitators. We responded to the needs of the MCD facilitators (i.e., the research participants), but at the same time our normative ideas about professional standards for MCD practice in general and for our own MCD practice, including a pool of approximately 50 trained MCD facilitators which we supervise, played a significant role. Our responsibilities, expertise and high standards regarding trained MCD facilitators influenced the development process. This led, for example, to our decision to include instructive parts with concrete guidance and suggestions for courses of action. Ultimately, we found a way to develop a tool that aims to maintain the quality of MCD, while at the same time maintaining our high standards for ethics support practice. This explains why our normativity is more prominent in the process of developing this tool in comparison to developed CES tools for health care practice or professionals.

Ultimately, the Confidentiality Compass is a tool that aims at reflection and is dialogical oriented, but it also contains parts with information on contexts and conceptual questions, and provides content with specific values ​​that could be considered. However, we did not include all aspects which were considered during the data gathering. For example, some research participants had questions about ‘the right of non-disclosure’ and protection of the 'freedom to maintain confidentiality'. Some wished to gain more clarity about the status of the role as MCD facilitator: what is expected from me in certain situations? In addition, some MCD facilitators requested a kind of moral oath for MCD facilitators, which states the responsibilities of a MCD facilitator and how to deal with confidentiality in MCD. The latter is not included because establishing rules of conduct would not fit with our view on ethics (support); rules and codes of conduct are contextual and only become relevant and meaningful after careful consideration and reflection [37]. We aimed for a tool that is relevant to all types of situations and moral challenges and we aimed for fostering individual moral reflection.

By using this tool, MCD facilitators are stimulated to individually reflect on their roles and responsibilities, and strengthen their competences in the role of MCD facilitator, especially regarding confidentiality in MCD. The Confidentiality Compass has been developed for individual reflection but can also be used for a collective dialogue. The tool can be used for professionalization, for example in a masterclass or training for MCD facilitators, as support for policymaking on confidentiality in other institutions, and during peer review sessions with experienced MCD facilitators. In that case the facilitator can use different elements of the tool to guide the conversation on an experience from practice. One can include certain steps or segments from the tool to the dialogue when the facilitator feels this would help the conversation on the topic.

### Part E

Based on the input from research participants we decided to add a part with specific support and instructions only for MCD facilitators that are affiliated to our institution (with more concrete instructions and contact details – part E). This part is still in development and not presented in this article. Part E consists of clear institutional agreements for MCD facilitators and provides clarity about their role and position within our institution. Moreover, it describes instructions for reporting incidents and recommends the path to follow when (considering) breaching confidentiality. Considering this aim and the responsibility of the organization of CES, which is situated in our department, part E resulted in a more normative approach towards (considering) breaching confidentiality.

As we described, the contextuality of a tool – how to translate the confidentiality agreements into concrete courses of actions – is of importance for developing and implementing a tool. The general tool (parts A-D) and the specific amendment (part E) have different target groups as users and are also different in terms of normativity. The more attention is paid to the specificity of a certain context, the more specific the tool is and therefore the more defined the target group that can use the tool. The lesson learned is that you can divide an ethics support tool into different parts in which you can distinguish between parts that are accessible to a wider audience and parts that are specifically intended for users in a specific context.

### Integrative ethics support

The development of the Confidentiality Compass can be seen as an example of integrative ethics support. This concept was introduced by Hartman et al. (2019) who present some lessons learned with developing ethics support in a responsive design with care professionals from transgender care practice [39]. Hartman (2020) describes five characteristics of the integrative ethics support approach, which is about positioning clinical ethics support in care practices, involving new perspectives, creating co-ownership of CES, paying attention to follow-up and developing innovative CES activities through emerging design [20]. The latter played an important role in our development process: the emerging design was not a preconceived idea but emerged throughout an iterative process between insights from research activities, ethics support (e.g. MCD sessions) and experiences in practice. The Confidentiality Compass can be used alongside the traditional forms of reflection during peer review sessions or training moments. By working together with MCD facilitators and involving them in the different steps of the development process, we aimed at creating ownership of both the developmental process and the content of this tool among the MCD facilitators and us as researchers.

### Strengths and limitations

One of the strengths of our research and development process was the participation of experts and potential users in different steps of the process. Participating in the research process through interviews, MCDs and focus groups provided MCD facilitators with the opportunity to critically reflect on their own experiences, beliefs and moral doubts, and those of others. The MCDs and focus groups provided opportunities for mutual exchange of ideas, critically questioning each other and to gain a better understanding of confidentiality in MCD. In this project we used MCD both as a form of ethics support, to enter into dialogue with MCD facilitators about their experienced moral challenges, and as a method for data collection. By using this approach we made a special contribution to the practice of MCD facilitators, and to the research and the development process of the ethics support tool.

Through the different methodologies and through co-creation, through research in cooperation with practice, broad support was created in the development of the Confidentiality Compass. The interplay of the different components of practice, research and ethics support provides the tool with a very strong foundation. The goal of this research was to promote reflection on questions of confidentiality in MCD facilitators, both through a joint developmental process and through the result of this process: the Confidentiality Compass.

The development process of this ethics support tool, as well the Confidentiality Compass itself, can be viewed as problem oriented. It started with troublesome questions from practice and tries to offer support in dealing with those questions. Future ethics support tools may have different starting points and goals. Furthermore, a limitation in the development process is the inclusion of limited perspectives. For example, we did not interview MCD participants, or policymakers, or a member of the management of the institution, while their interests and ideas on (potentially breaching) confidentiality are of great importance, for example when it comes to handling medical mistakes or a certain climate of communication within an institution. Time constraints and limited resources, in the context of Covid restrictions, forced us to make choices. We would recommend to further develop the tool and include more relevant perspectives. Another limitation in the design of the tool is that we did not address all the needs mentioned by MCD facilitators in response to questions concerning confidentiality in MCD. The Confidentiality Compass does not provide any legal clarification, which is beyond our expertise, but could be part of a next (advanced) version of the tool. Lastly, we did not yet evaluate the usage of the Confidentiality Compass in practice in a structured way. One recommendation for future research would be to implement the Confidentiality Compass in a specific context and evaluate the relevance and practicality of the tool, and collect topics for improvement. A further research focus could be to investigate whether the tool is useful for participants in MCD or health care professionals dealing with confidentiality questions: what elements need to be altered in order to make the tool useful for these other groups?

## Conclusions

Based on MCD facilitators’ concrete CES experiences and reported needs for ethics support, we started to investigate the theme of confidentiality in MCD, the related moral challenges, and possible ways of developing a CES tool. This paper is relatively unique in that it describes the development process and content of the developed ethics support tool. During the process we learned that ethics support tools can be seen as merely heuristic instruments and we therefore recommend considering additional characteristics other than only (self-)reflective questions. For example, adding informative parts about the concept, a description of best practices collected through research, the relevance of virtues (in addition to values and norms), some central weighing principles, and providing different proposed courses of actions. During the development process we continuously reflected on the normativity in the process and content of the tool. This will remain a challenge in future tool development processes and other innovations in clinical ethics support.

The tool was developed for individual reflection but can also be used for collective dialogue. Besides using the tool when MCD facilitators experience a moral challenge (i.e., demand driven), the tool can also be pro-actively used in training and peer review session for (trained) MCD facilitators, or as support for policy making regarding confidentiality. Finally, we recommend the further development of this ethics support tool, taking into account its broader usefulness and contribution in clinical practice: after implementation, the use and value of the tool should be further evaluated. This ethics support tool and its content were aimed at MCD facilitators who are confronted with moral challenges related to confidentiality, but both the developmental process and the content of the tool may also be applicable in and for other CES contexts where either confidentiality is important or where one aims to develop an ethics support tool. In this way, the professionalization of ethics support services can further evolve.

## Data Availability

The data generated and/or analyzed during the current study are not publicly available but are available from the corresponding author on reasonable request.
